# Supplementary material for: Phellinus linteus Mycelia Extracts Show Potent Antiviral and Immunomodulatory Effects in H1N1 Influenza Virus-Infected Mice
Source: Foods. 2025 Nov 26;14(23):4047. doi: 10.3390/foods14234047 (PMC12692646; doi:10.3390/foods14234047)
Supplement: Supplementary file 1 [file foods-14-04047-s001.zip › Supplementary Figure Legends.pdf]

Figure Legends

Figure S1. Effects of *P. linteus* extracts on MDCK cell viability. MDCK epithelial cells were treated with (A) water extract (PLw) or (B) ethanol extract (PLe) of *P. linteus* mycelia for 48 hours. Cell viability was determined using MTS assay and expressed as a percentage relative to untreated control cells. Values represent mean ± SD from three independent experiments. Asterisks denote statistically significant differences compared to control group ($p < 0.05$).

Figure S2. Plaque inhibition effects of *P. linteus* mycelial extracts on H1N1-infected MDCK cells. The antiviral activity was evaluated using a plaque reduction assay. Representative images show the effects of (A) *P. linteus* water extract (1000 μg/mL) and (B) ethanol extract (250 μg/mL) under preventive, co-treatment, and therapeutic conditions.

Figure S3. Virulence of A/WSN/33 (H1N1) at different doses in 6-week-old BALB/c mice. Six-week-old BALB/c mice were exposed to A/WSN/33 (H1N1) via aerosol inhalation at doses of 1×10^4, 1×10^5, 1×10^6, and 1×10^7 PFU/mL. The mice were monitored daily for 14 days post-inoculation to assess survival rates. Values are presented as mean ± SD for each group (n=5).

Figure S4. Analysis of immune cell populations in bronchoalveolar lavage fluid (BALF) following treatment with *P. linteus* mycelial extracts. Flow cytometry analysis quantified the percentages of immune cells in BALF, including (A) helper T cells (CD3+CD4+), (B) cytotoxic T cells (CD3+CD8+), and (C) NK cells (NK1.1+).

Figure S1

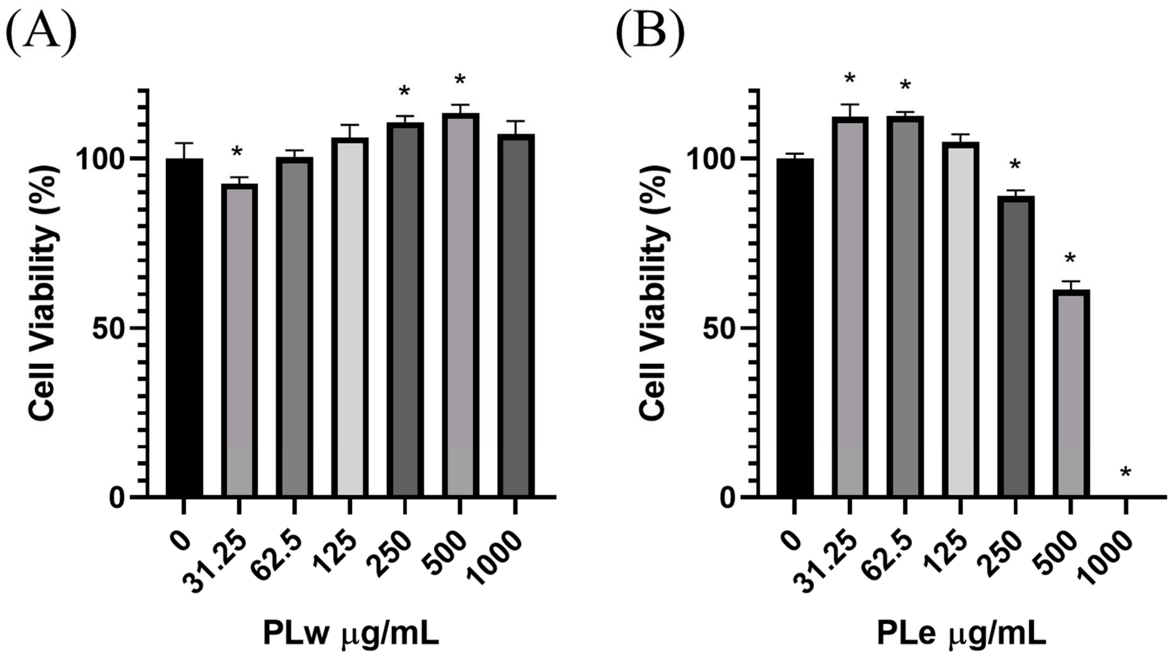

(A)
Cell Viability (%)
0
50
100
*
*
*
0
31.25
62.5
125
250
500
1000
PLw μg/mL
(B)
Cell Viability (%)
0
50
100
*
*
*
*
*
0
31.25
62.5
125
250
500
1000
PLe μg/mL

Figure S2

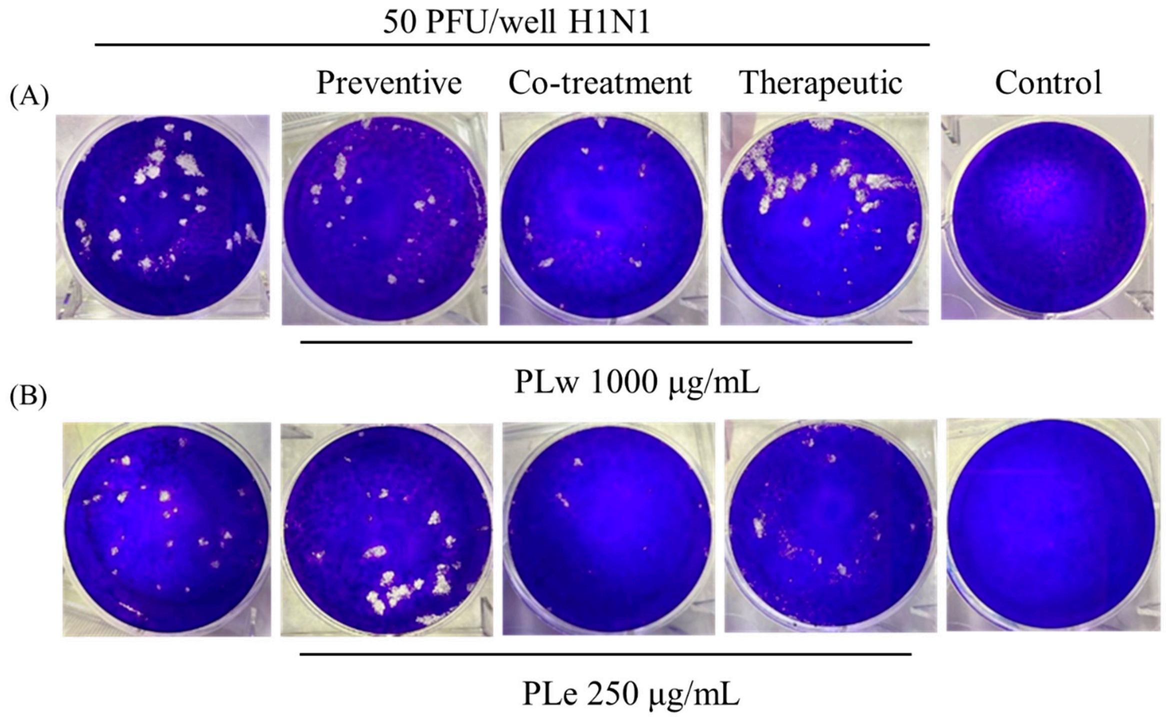
50 PFU/well H1N1
(A)
Preventive
Co-treatment
Therapeutic
Control
PLw 1000 μg/mL
(B)
PLe 250 μg/mL

Figure S3

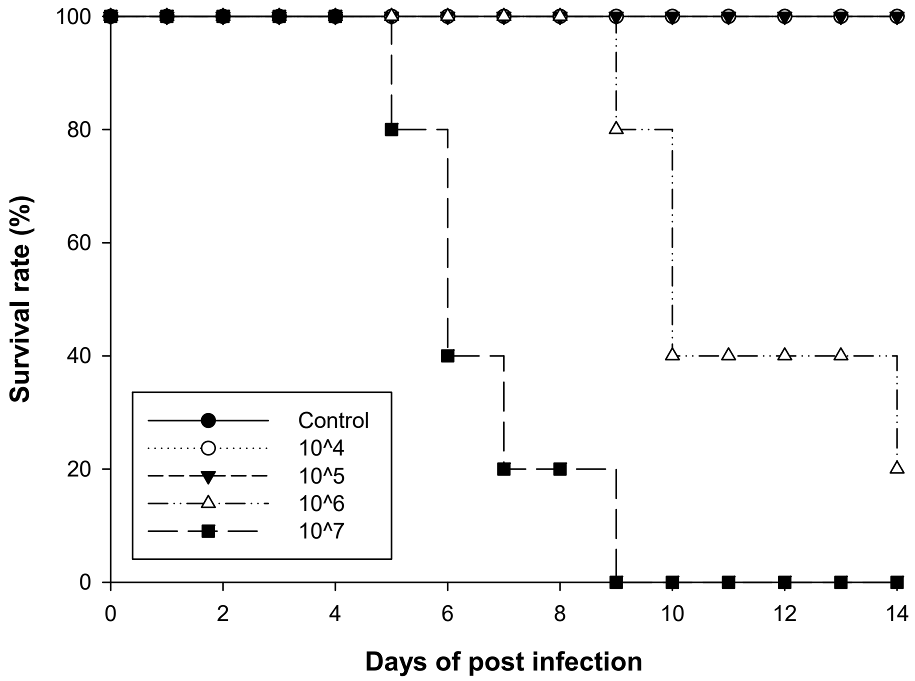
100
80
60
40
20
0
Survival rate (%)
0
2
4
6
8
10
12
14
Days of post infection
Control
10^4
10^5
10^6
10^7

Figure S4

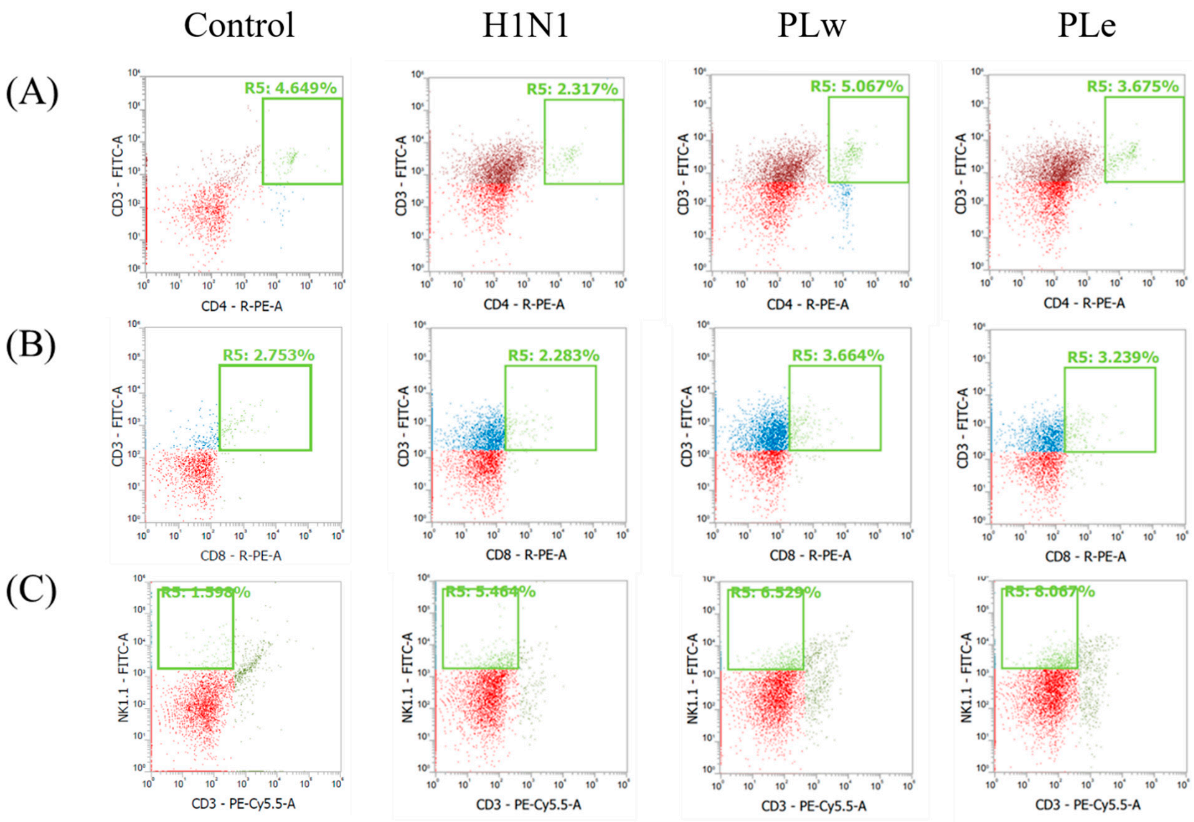

Control
H1N1
PLw
PLe
(A)
R5: 4.649%
R5: 2.317%
R5: 5.067%
R5: 3.675%
CD3 - FITC-A
CD4 - R-PE-A
(B)
R5: 2.753%
R5: 2.283%
R5: 3.664%
R5: 3.239%
CD3 - FITC-A
CD8 - R-PE-A
(C)
R5: 1.598%
R5: 5.464%
R5: 6.529%
R5: 8.067%
NK1.1 - FITC-A
CD3 - PE-Cy5.5-A
