# Supplementary material for: Phellinus linteus Mycelia Extracts Show Potent Antiviral and Immunomodulatory Effects in H1N1 Influenza Virus-Infected Mice
Source: Foods. 2025 Nov 26;14(23):4047. doi: 10.3390/foods14234047 (PMC12692646; doi:10.3390/foods14234047)
Supplement: Supplementary file 1 [file foods-14-04047-s001.zip › Table S.pdf]

Table S1. Primers used in this study

Target Gene	Sequence 5'-3'	Annealing Temperature	Size (bp)
Hemagglutinin	F: TAACCTGCTCGAAGACAGAC<br>R: AGAGCCATCCGGTGATGTTA	60°C	95
IL-6	F: TCCATCCAGTTGCCTTCTTG<br>R: TTTCTCATTTCCACGATTTCCC	57°C	175
TNF-α	F: GGTGTTCATCCATTCTCTAC<br>R: CCCAGCATCTTGTGTTTC	53°C	177
IFN-γ	F: AGCGGCTGACTGAACTCAGATTGTAC<br>R: GTCACAGTTTTCAGCTGTATAGGG	60°C	247
GAPDH	F: GCGACTTCAACAGCAACTC<br>R: GGTCCAGGGTTTCTTACTCC	57°C	171

IL-6: Interleukin 6; TNF-α: Tumor Necrosis Factor α; IFN-γ: Interferon Gamma; GAPDH: glyceraldehyde-3-phosphate dehydrogenase

Table S2. Chemical composition of PLe

Name	Formula	RT	Area	m/z	Extract Mass	Accurate mass	Score
Caffeic acid	C9 H8 O4	2.993	282566	179.03496	180.04224	180.04226	96.66
Hispolon	C12 H12 O4	3.654	147895	219.0662	220.07343	220.07356	99.18
4-(3,4-dihydroxyphenyl)but-3-en-2-one	C10 H10 O3	3.654	79461	177.0555	178.06293	178.06299	94.08
Fasciculine B	C24 H16 O10	3.776	516449	523.08765	464.07392	464.07435	89.47
Inotilone	C12 H10 O4	3.78	273932	219.06523	218.05793	218.05791	99.16
Fasciculine A	C24 H16 O9	3.794	286208	507.09266	448.07889	448.07943	94.32
Phellifuropyranone A	C21 H14 O7	3.811	61427	423.07198	378.07377	378.07395	94.26
Phelligridin C	C20 H12 O7	3.828	77340	423.07205	364.05817	364.0583	96.31
Hispidin	C13 H10 O5	4.037	24487421	245.04574	246.05299	246.05282	96.65
Phelligridin A	C13 H8 O6	4.058	52556	283.02074	260.03194	260.03209	84.07
Phelligridin D	C20 H12 O8	4.354	12689	381.06046	380.05349	380.05322	82.48
Bisnoryangonin	C13 H10 O4	4.455	5521416	229.05089	230.05814	230.05791	99.66
Hypholomine B	C26 H18 O10	4.925	8863117	489.08253	490.08979	490.09	88.72
Hypholomine A	C26 H18 O9	5.239	531404	473.08766	474.09485	474.09508	99.29
Inonotic acid methyl ester	C14 H14 O6	5.691	1108810	277.07163	278.07826	278.07904	99.57
Pinillidine	C28 H22 O10	6.701	3762510	517.11367	518.12096	518.1213	99.69
Methylinoscavin A	C26 H20 O9	6.701	158202	535.12411	476.11095	476.11073	88.69
Squarrosidine	C27 H20 O9	7.485	37517	487.10225	488.11011	488.11073	91.53

Reprint from “Phellinus linteus mycelia extract in COVID-19 prevention and identification of its key metabolic compounds profiling using UPLC-QTOF-MS/MS spectrometry” by Li et al., Fitoterapia 171 (2023) 105695.
