# Supplementary figures and images for: Phellinus linteus Mycelia Extracts Show Potent Antiviral and Immunomodulatory Effects in H1N1 Influenza Virus-Infected Mice
Source: Foods. 2025 Nov 26;14(23):4047. doi: 10.3390/foods14234047 (PMC12692646; doi:10.3390/foods14234047)

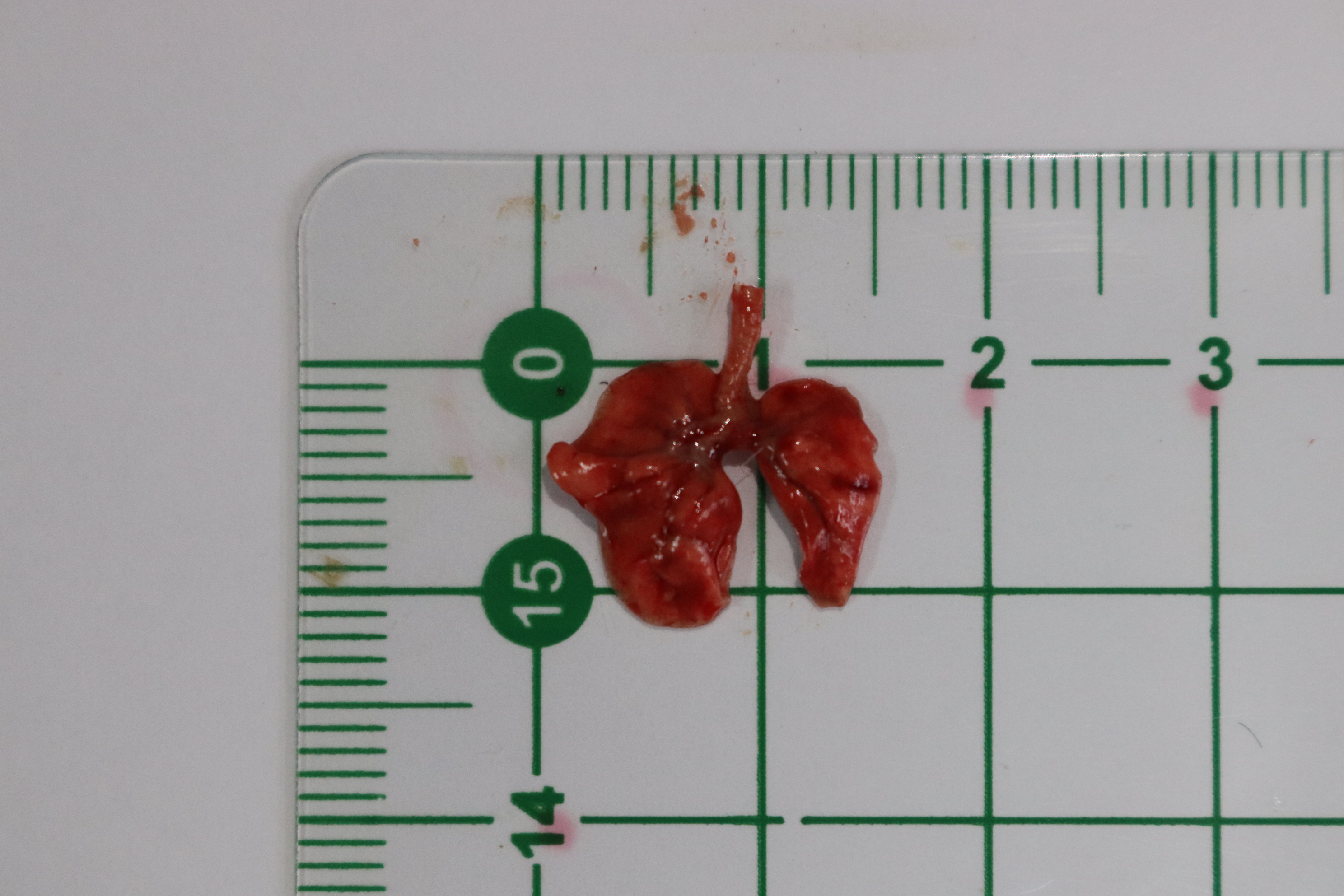

Supplement: Supplementary file 1 [file foods-14-04047-s001.zip › Individual Figures/Figure 4A/Control.JPG]

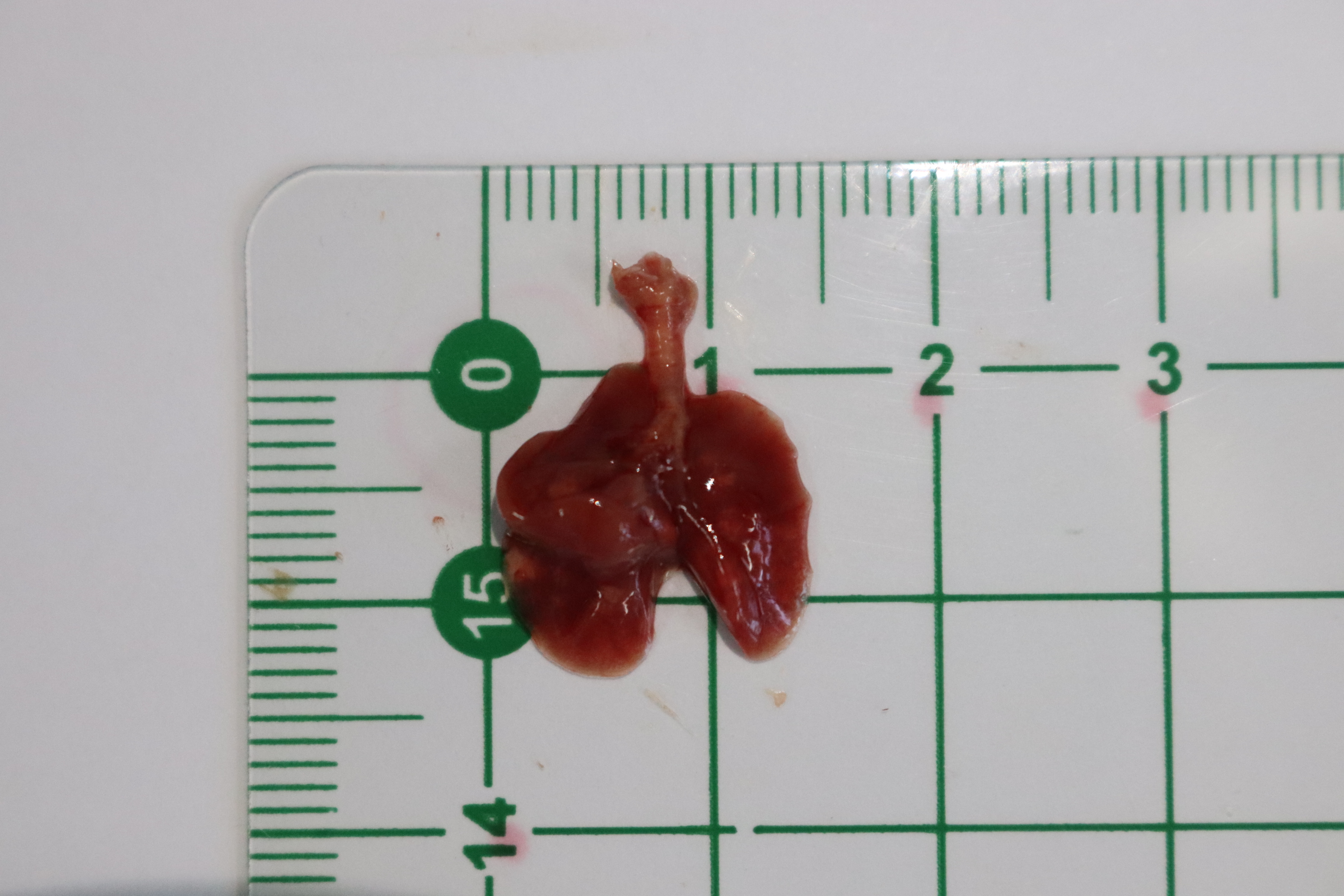

Supplement: Supplementary file 1 [file foods-14-04047-s001.zip › Individual Figures/Figure 4A/H1N1.JPG]

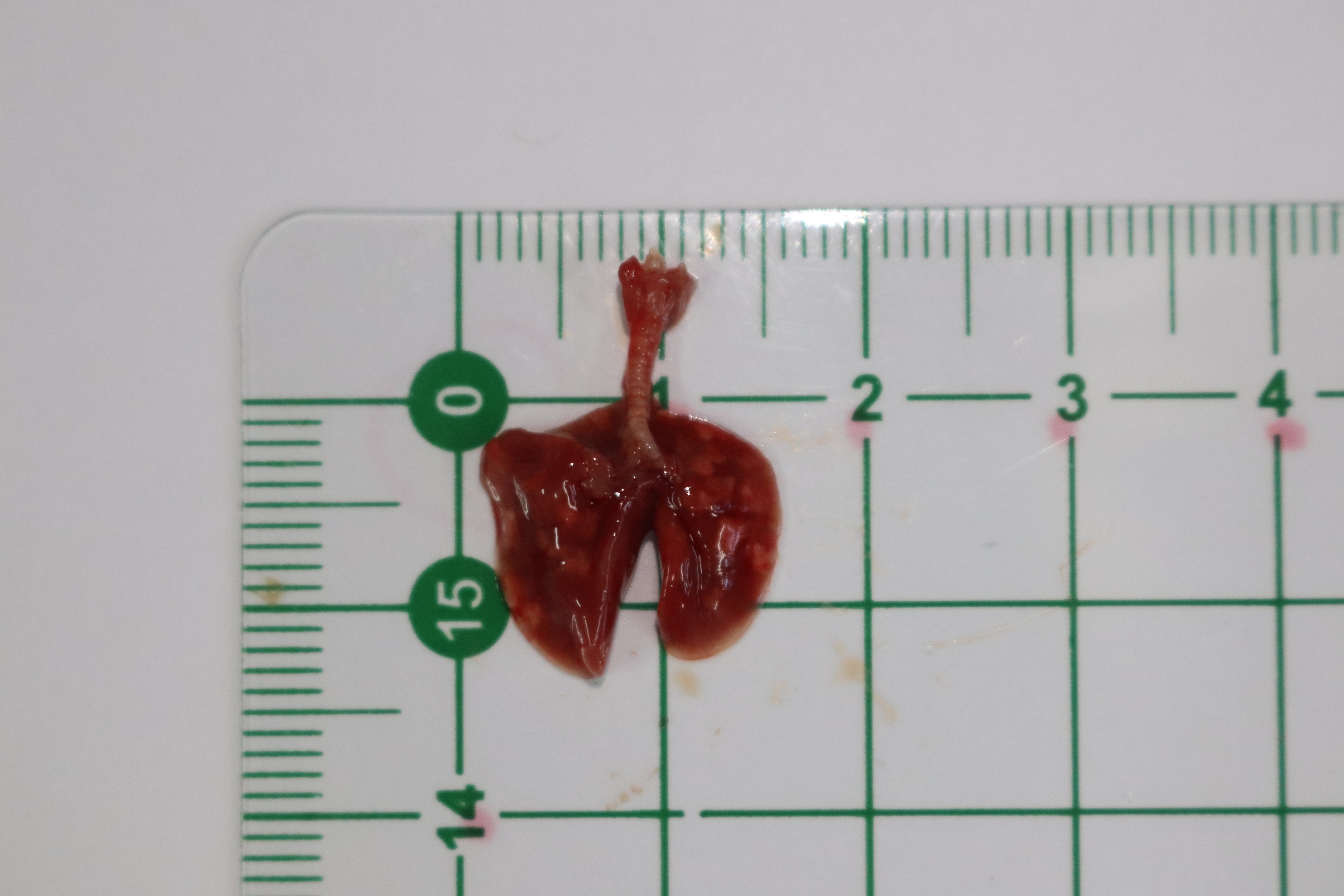

Supplement: Supplementary file 1 [file foods-14-04047-s001.zip › Individual Figures/Figure 4A/PL.JPG]

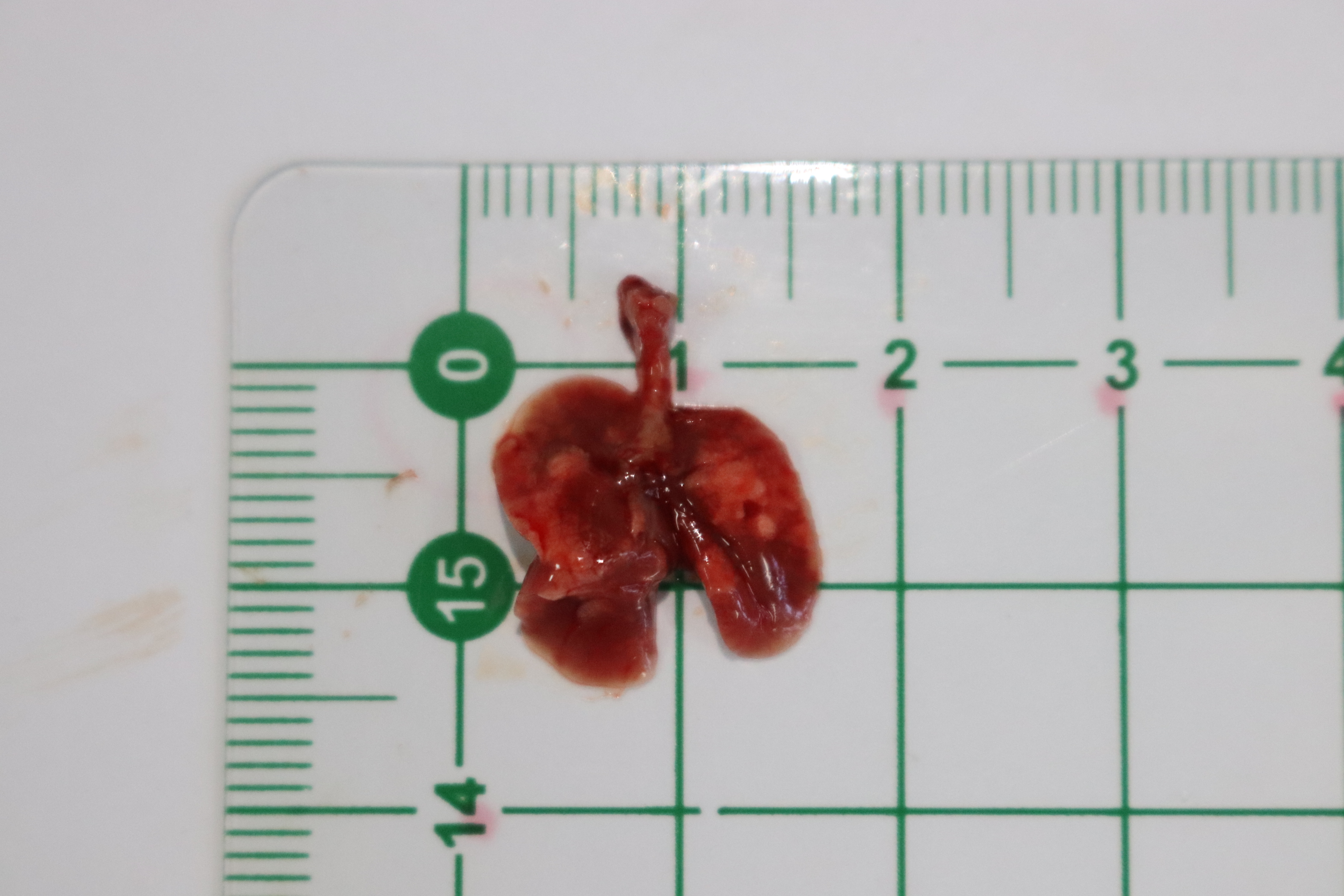

Supplement: Supplementary file 1 [file foods-14-04047-s001.zip › Individual Figures/Figure 4A/PLe.JPG]

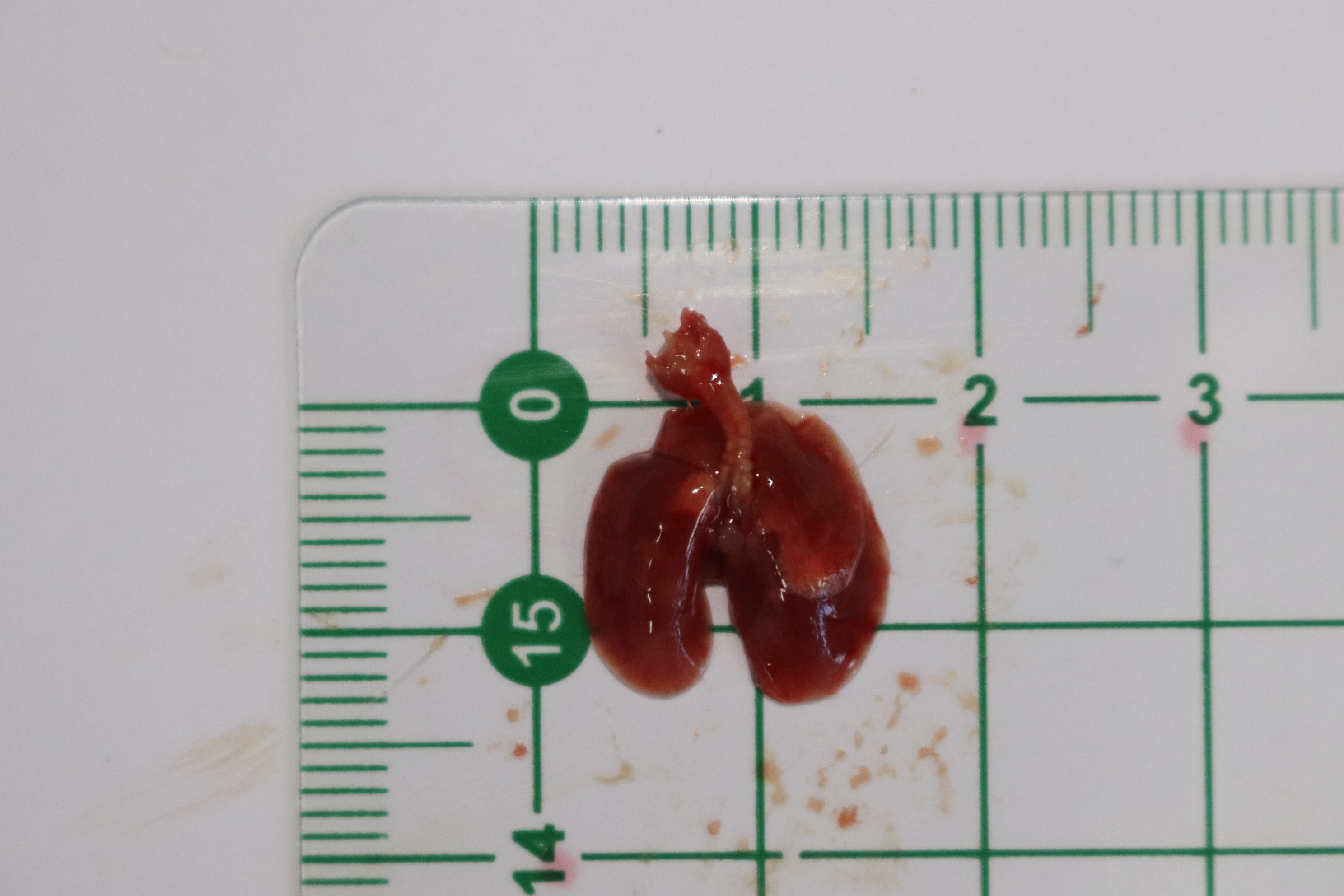

Supplement: Supplementary file 1 [file foods-14-04047-s001.zip › Individual Figures/Figure 4A/PLw.JPG]

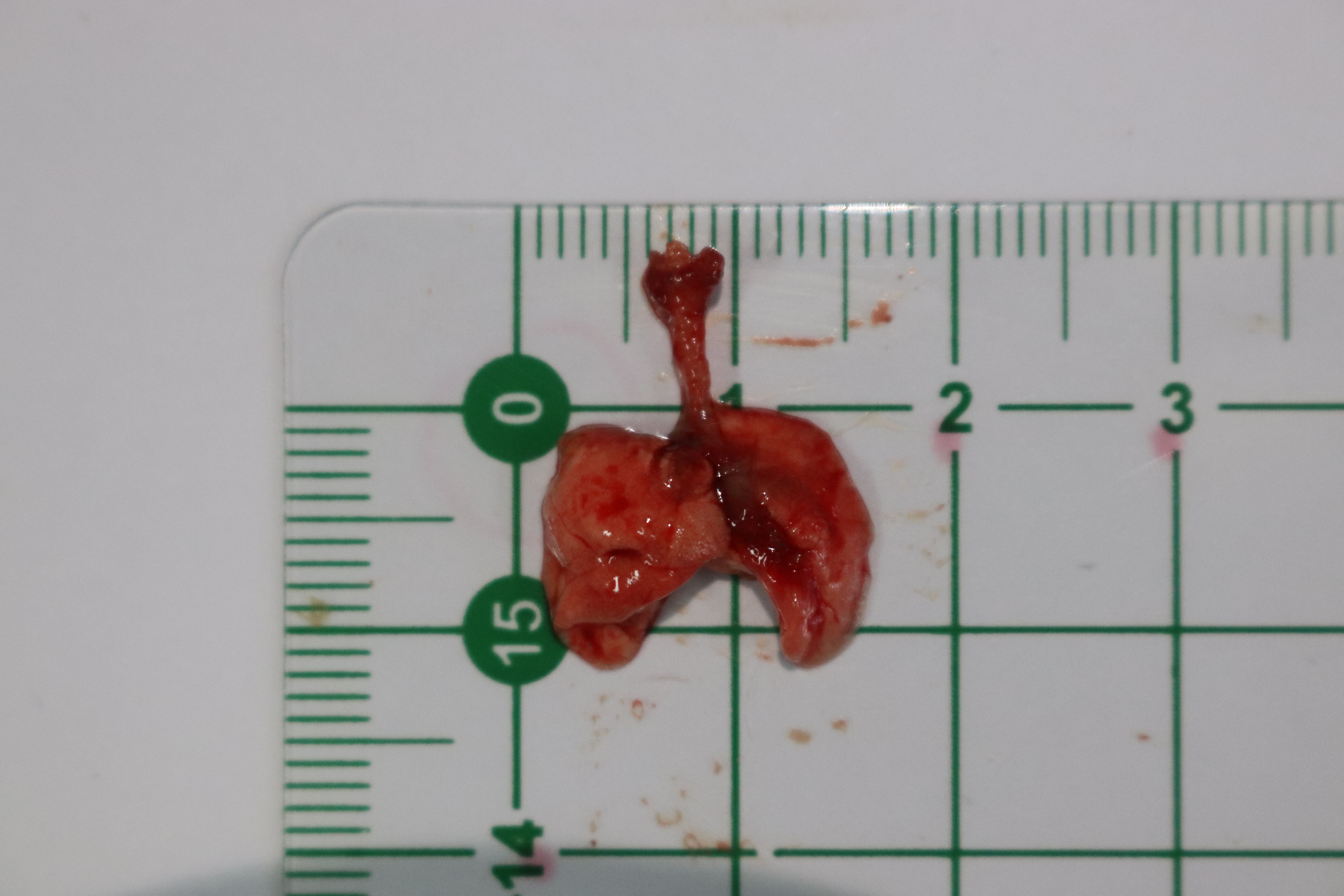

Supplement: Supplementary file 1 [file foods-14-04047-s001.zip › Individual Figures/Figure 4A/Tamiflu.JPG]

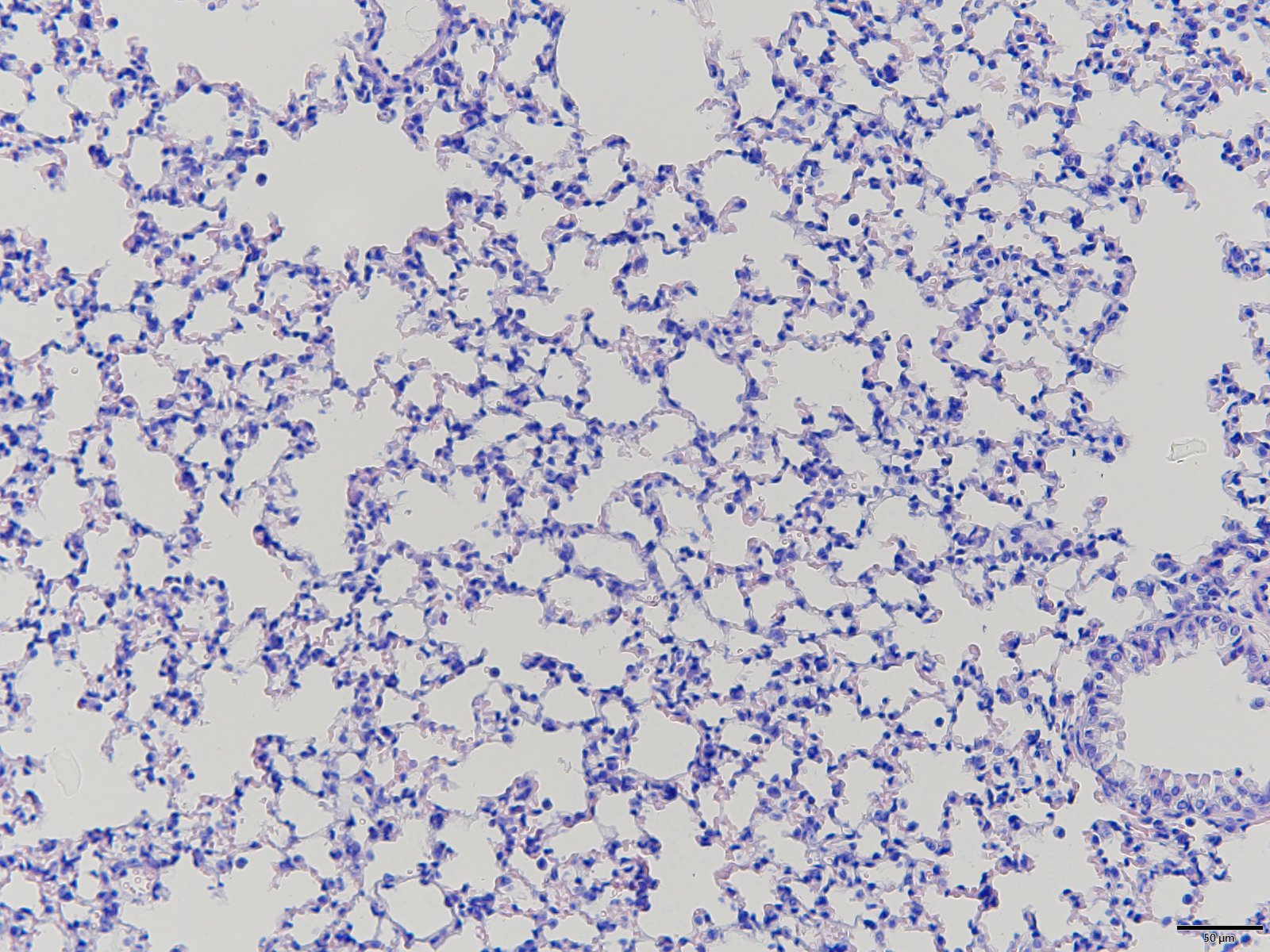

Supplement: Supplementary file 1 [file foods-14-04047-s001.zip › Individual Figures/Figure 4C/Control.jpg]

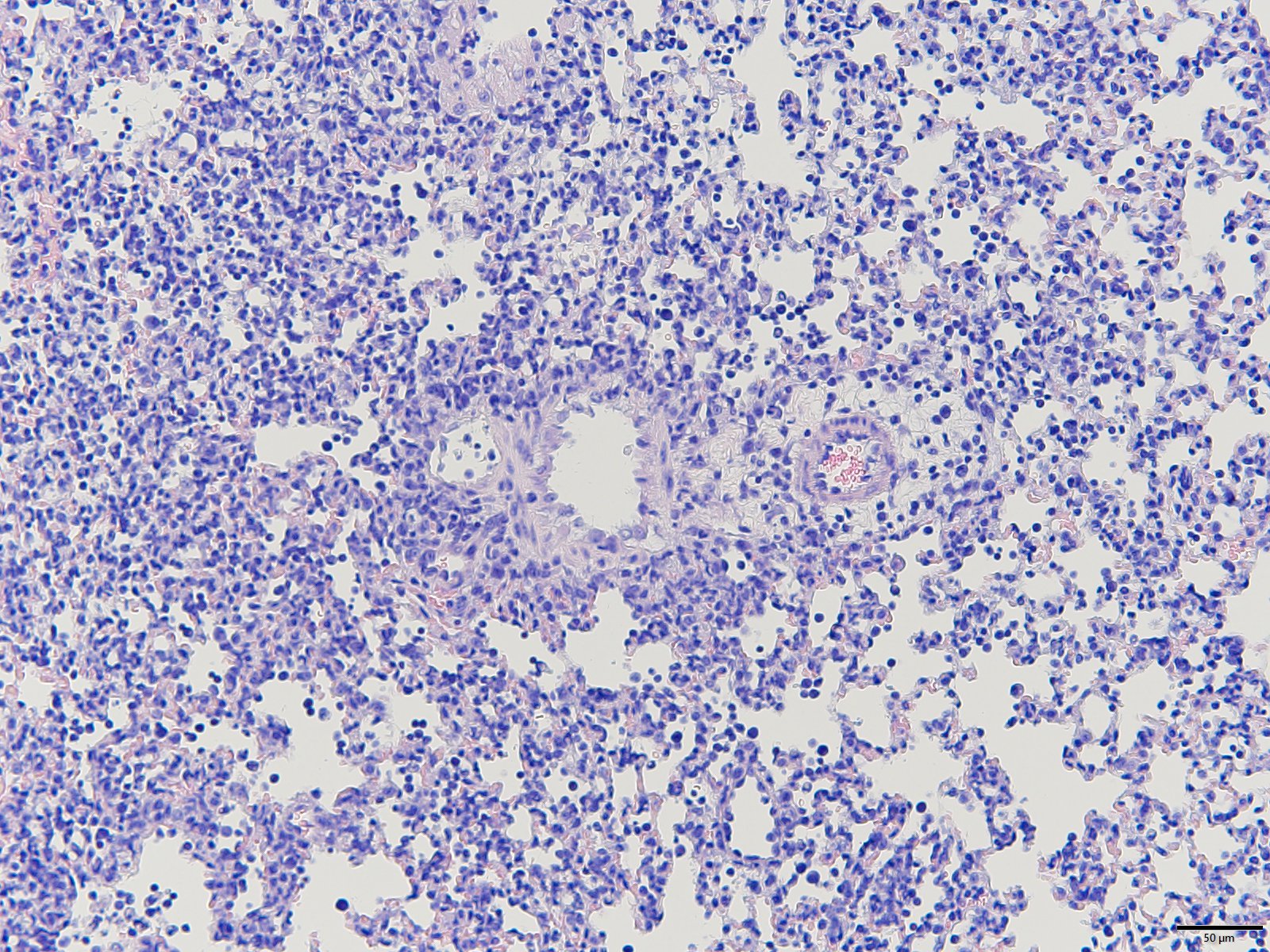

Supplement: Supplementary file 1 [file foods-14-04047-s001.zip › Individual Figures/Figure 4C/H1N1.jpg]

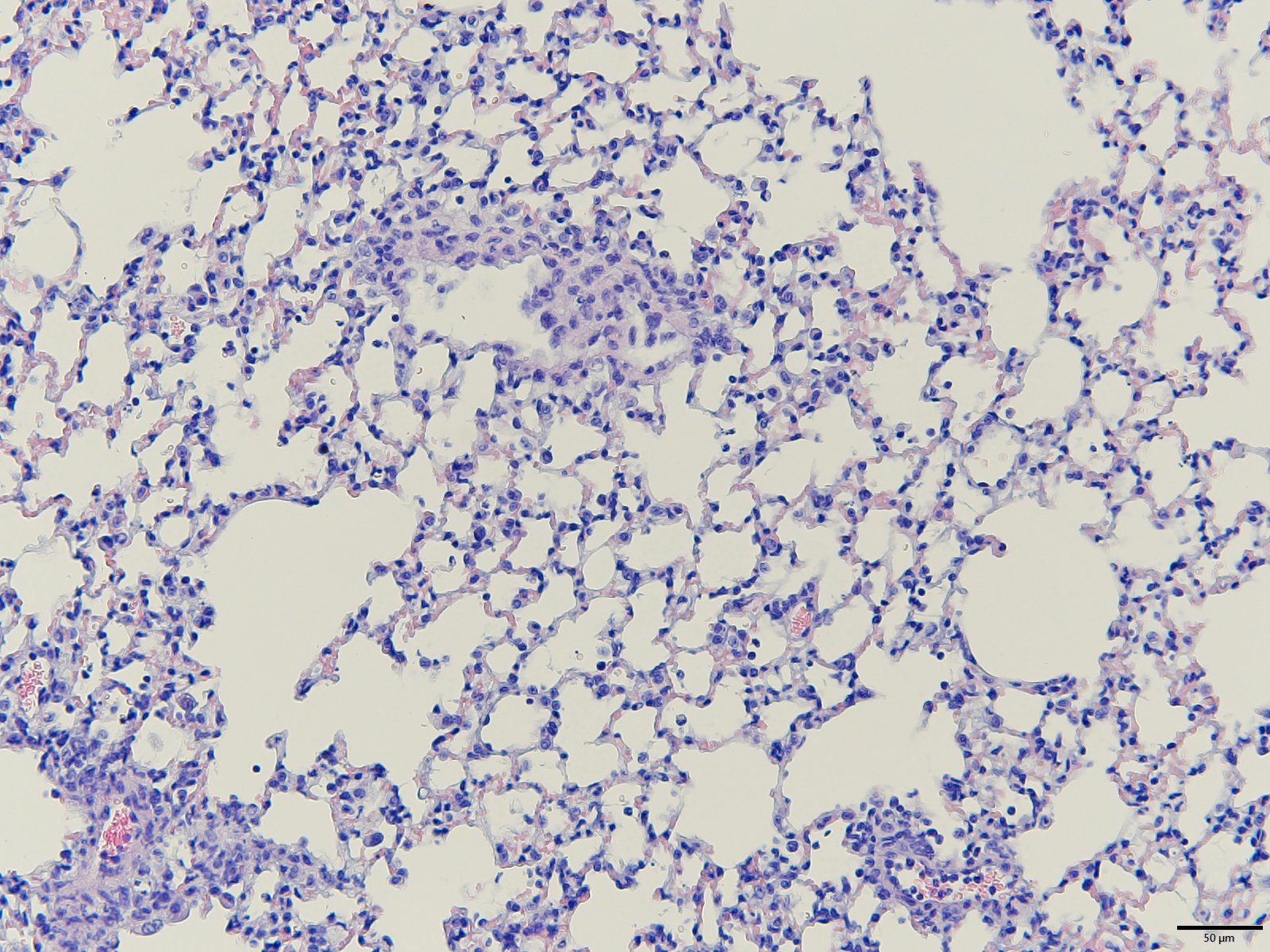

Supplement: Supplementary file 1 [file foods-14-04047-s001.zip › Individual Figures/Figure 4C/PL.jpg]

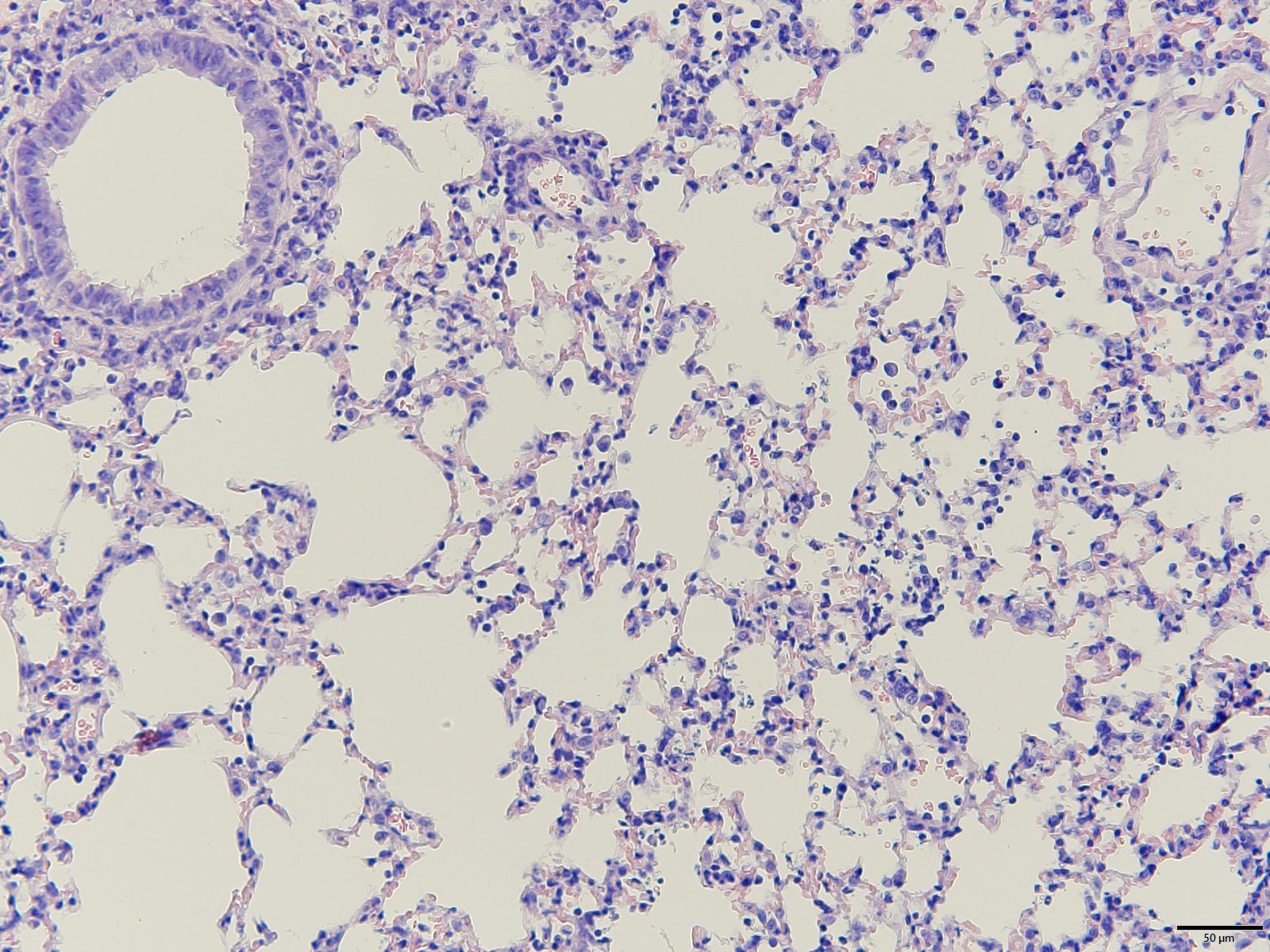

Supplement: Supplementary file 1 [file foods-14-04047-s001.zip › Individual Figures/Figure 4C/PLe.jpg]

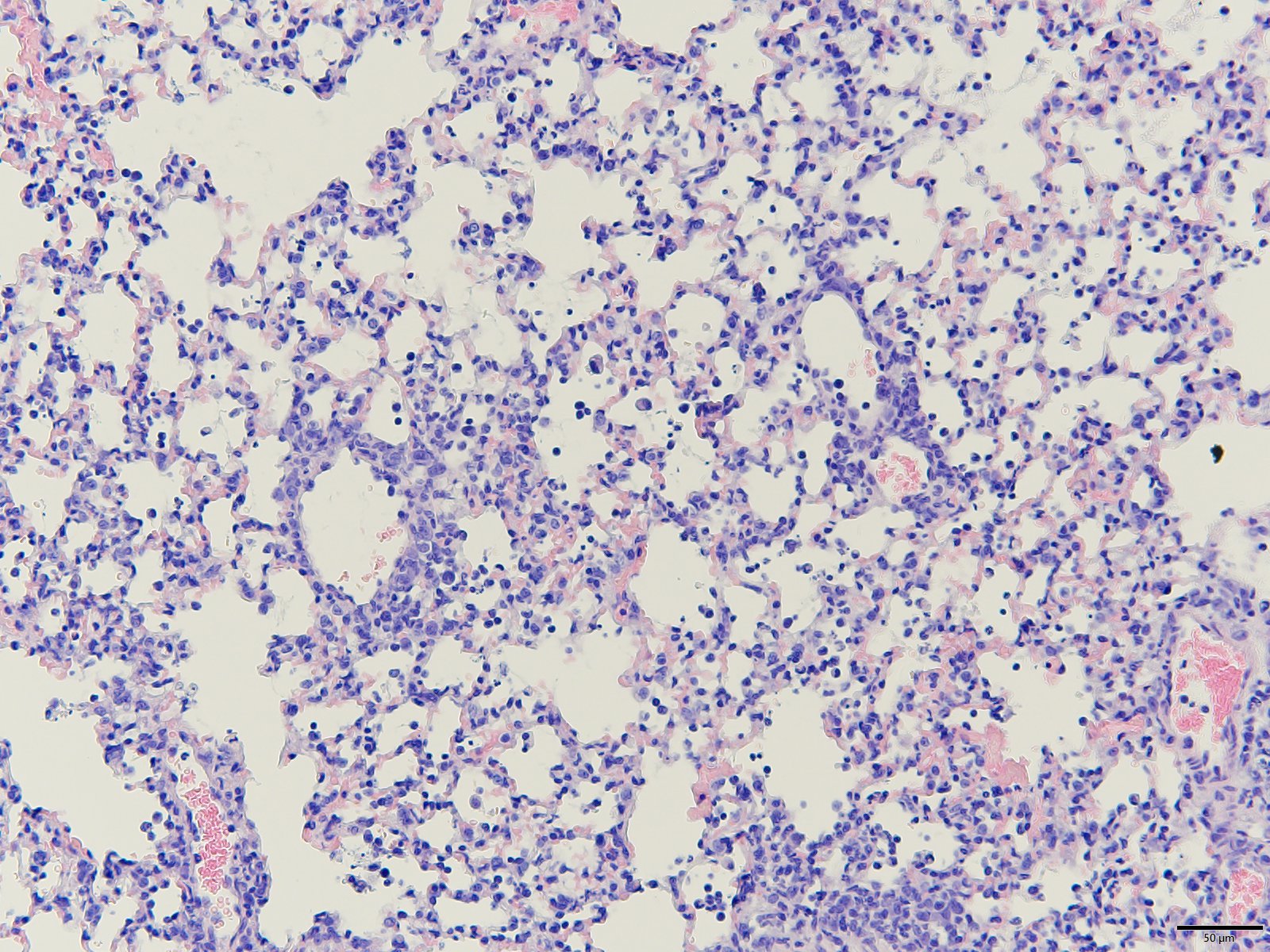

Supplement: Supplementary file 1 [file foods-14-04047-s001.zip › Individual Figures/Figure 4C/PLw.jpg]

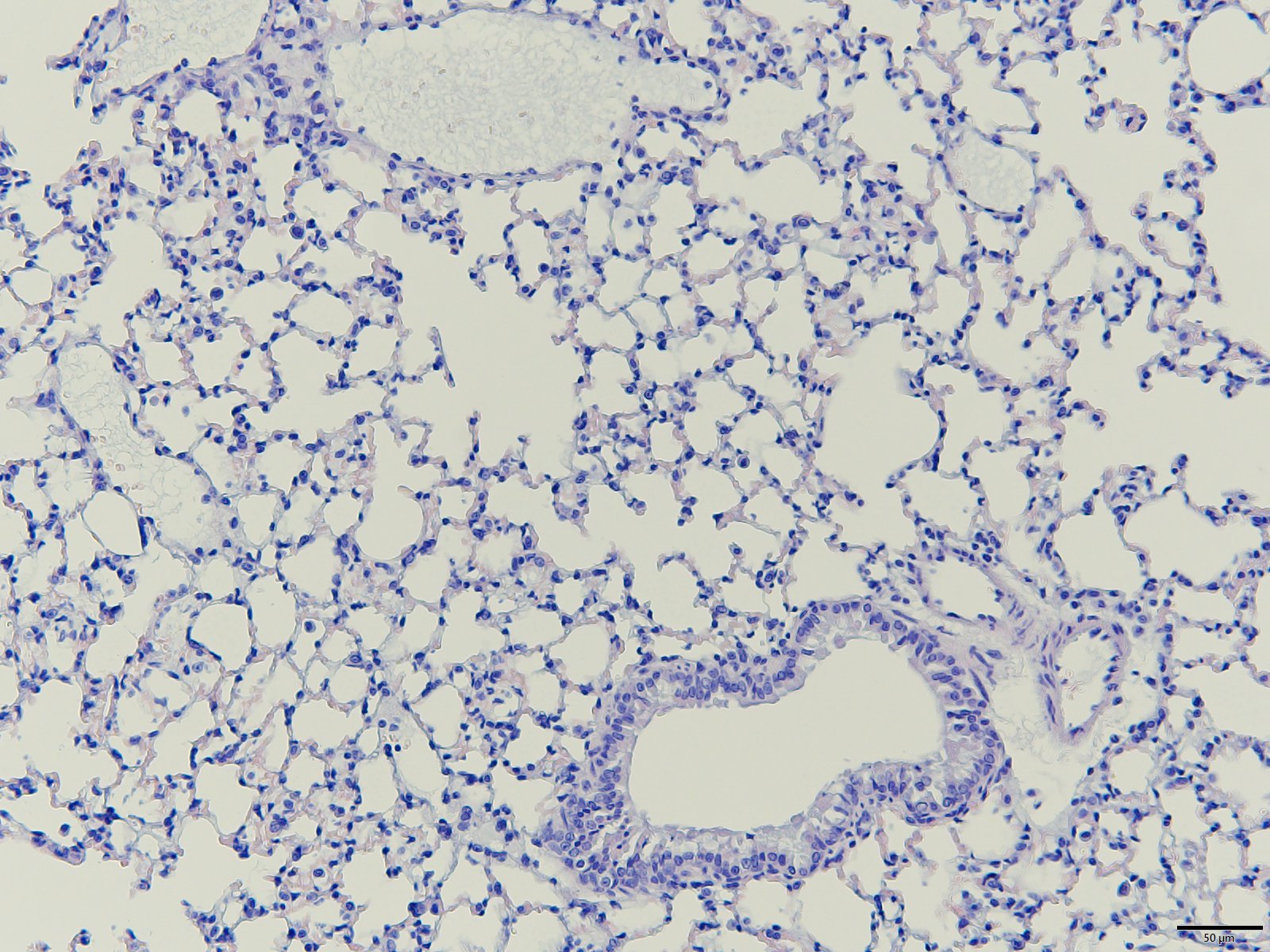

Supplement: Supplementary file 1 [file foods-14-04047-s001.zip › Individual Figures/Figure 4C/Tamiflu.jpg]

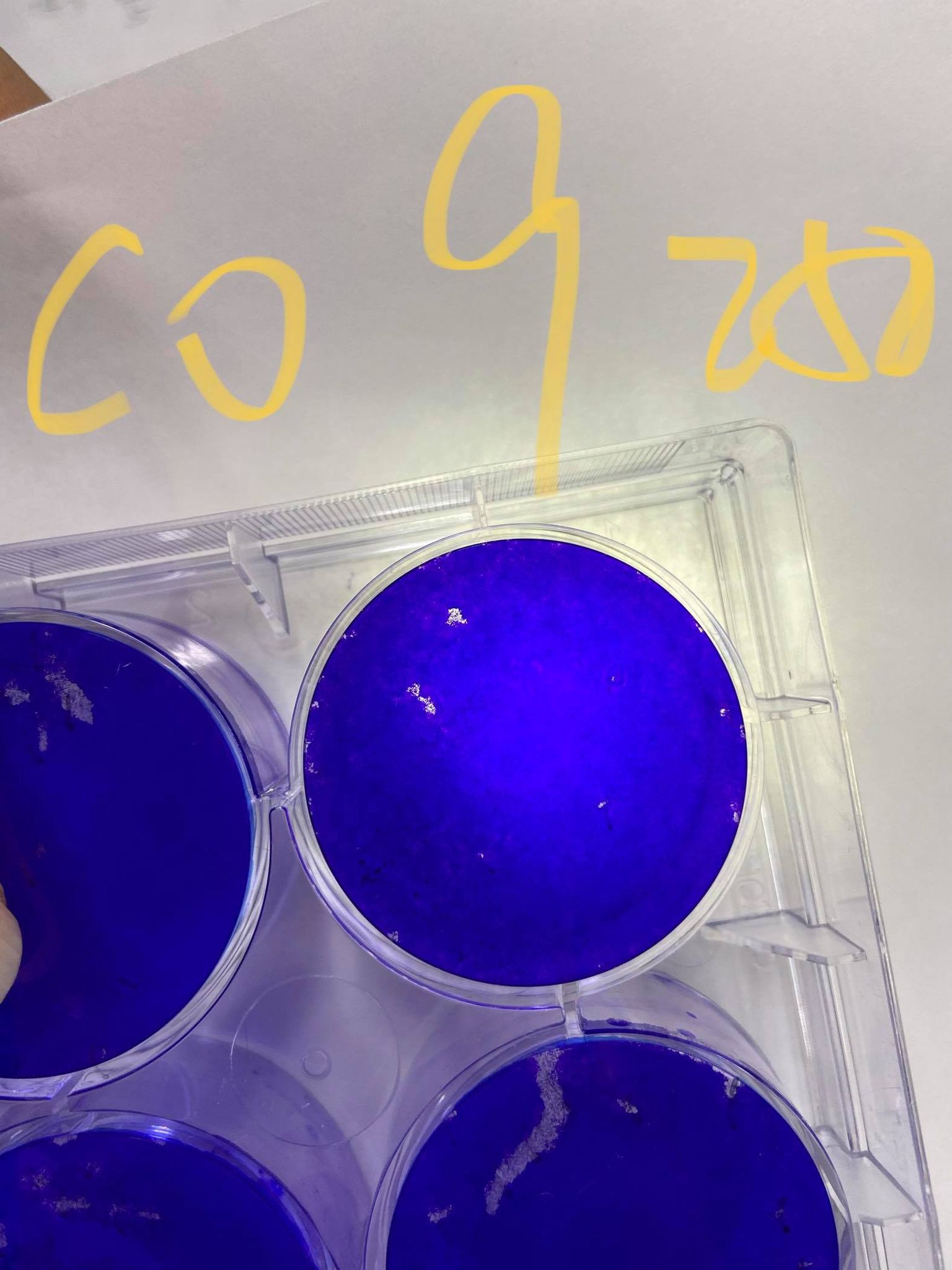

Supplement: Supplementary file 1 [file foods-14-04047-s001.zip › Individual Figures/Figure S2/PLe/Co PLe 250.jpg]

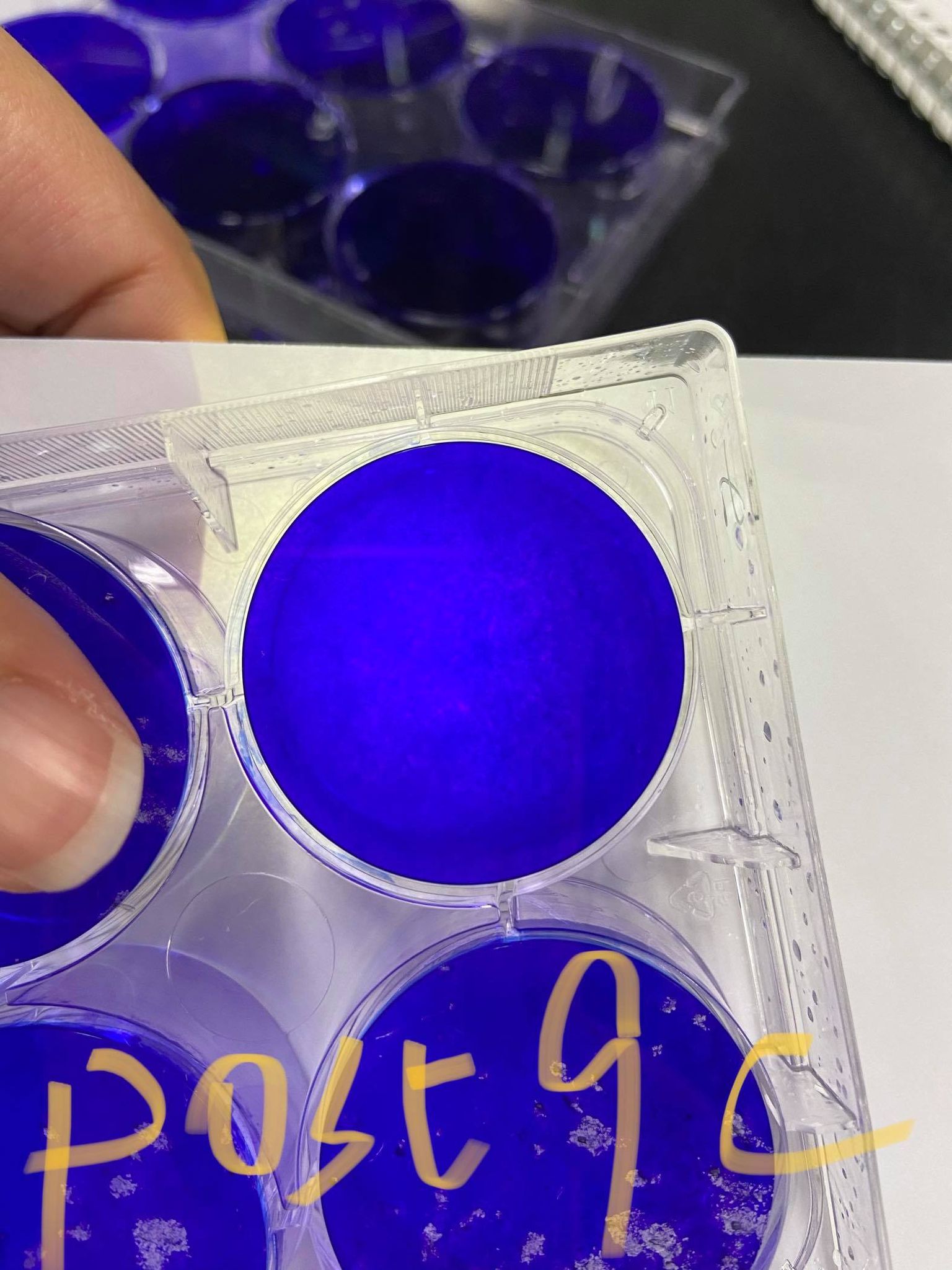

Supplement: Supplementary file 1 [file foods-14-04047-s001.zip › Individual Figures/Figure S2/PLe/Control .jpg]

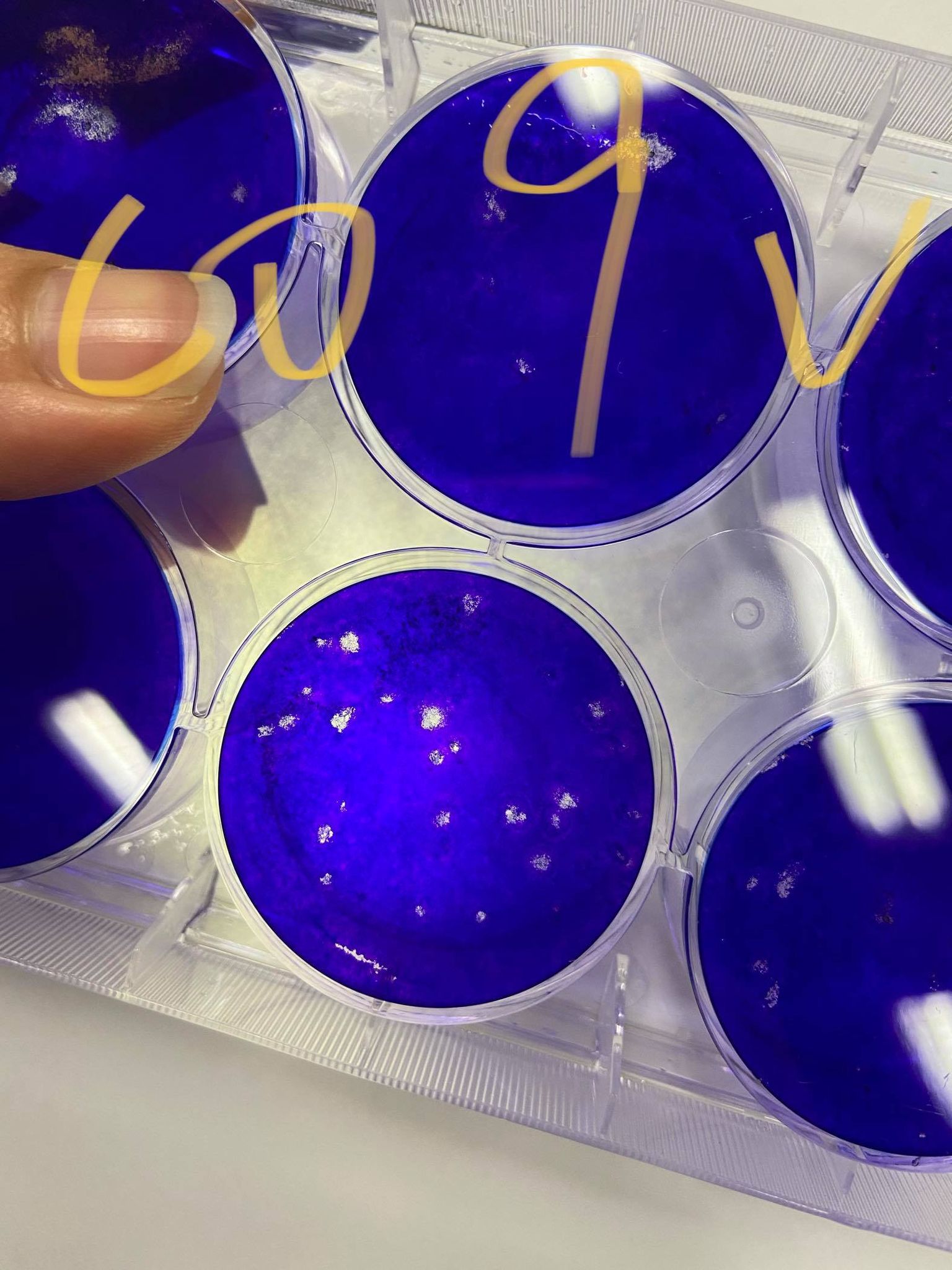

Supplement: Supplementary file 1 [file foods-14-04047-s001.zip › Individual Figures/Figure S2/PLe/PLe V.jpg]

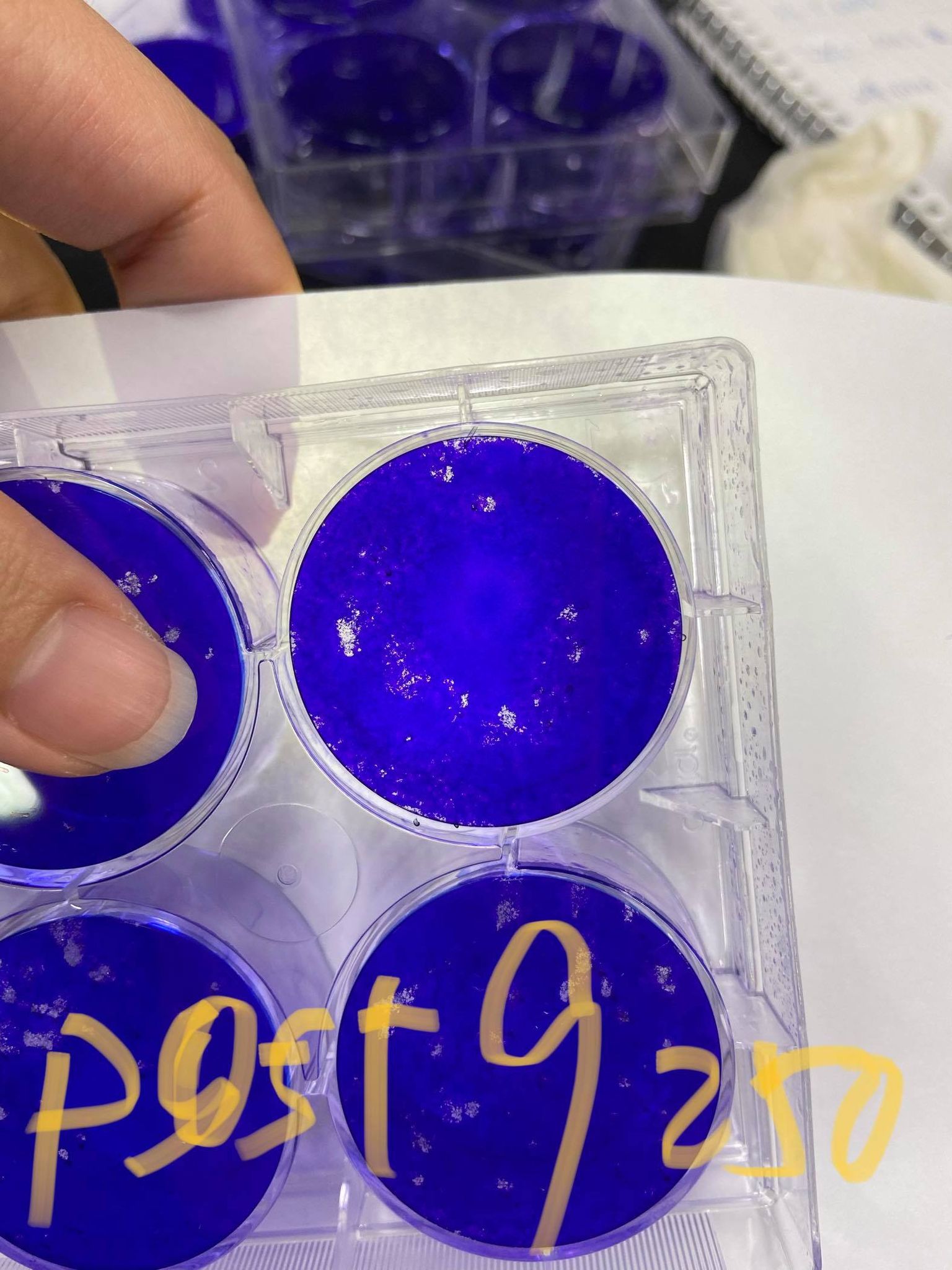

Supplement: Supplementary file 1 [file foods-14-04047-s001.zip › Individual Figures/Figure S2/PLe/Post PLe 250.jpg]

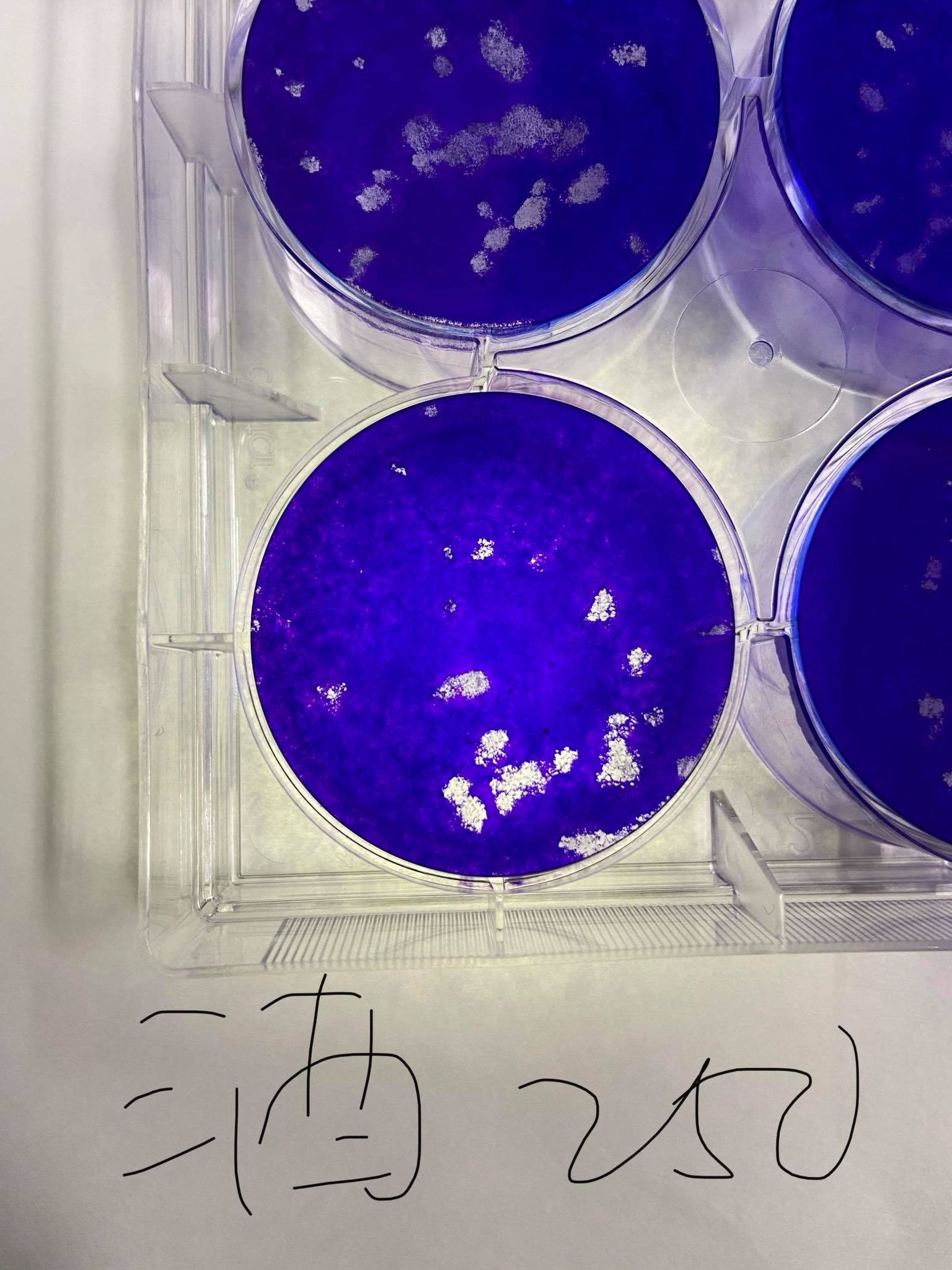

Supplement: Supplementary file 1 [file foods-14-04047-s001.zip › Individual Figures/Figure S2/PLe/Pre PLe 250.jpg]

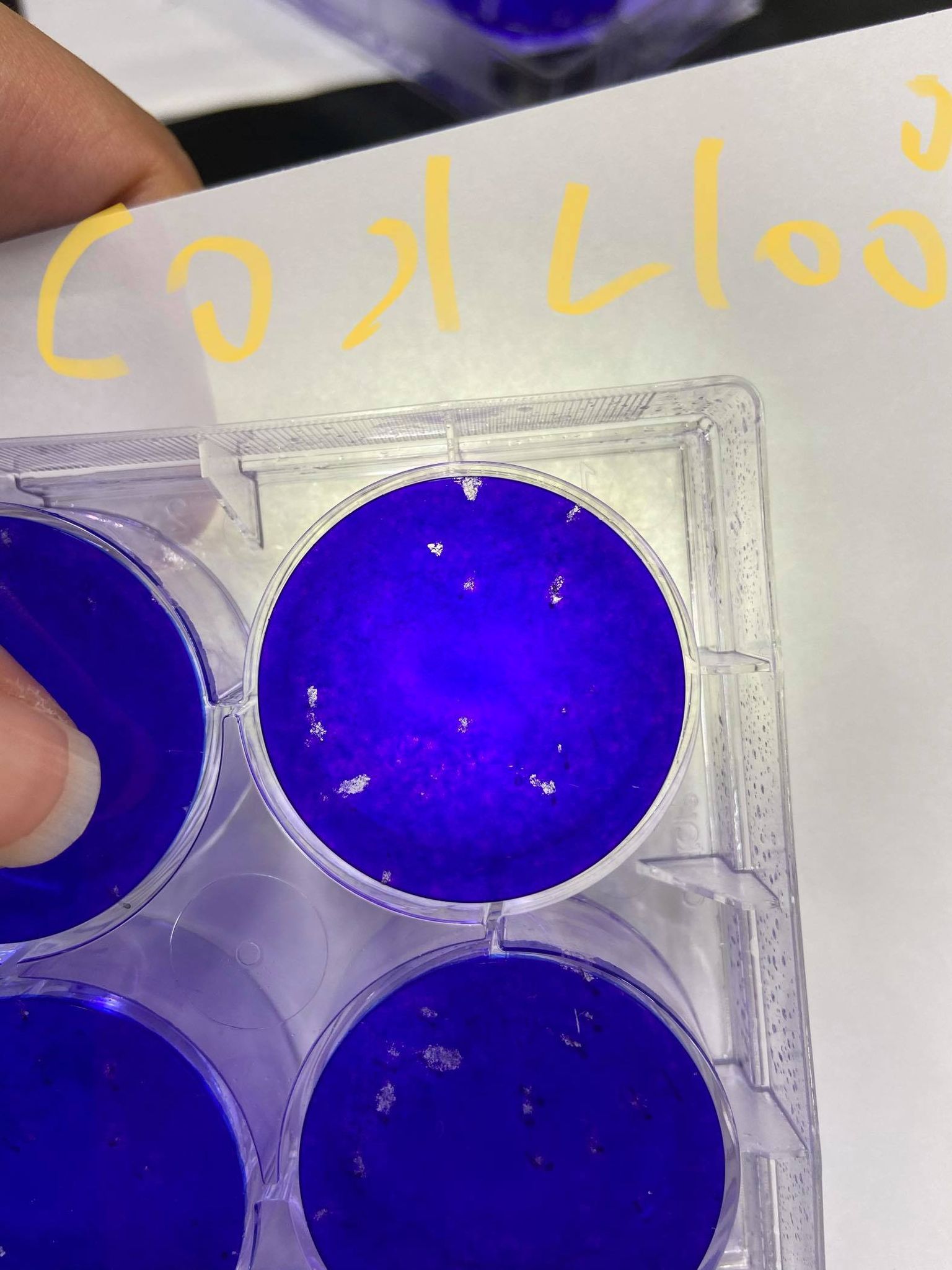

Supplement: Supplementary file 1 [file foods-14-04047-s001.zip › Individual Figures/Figure S2/PLw/Co PLw 1000.jpg]

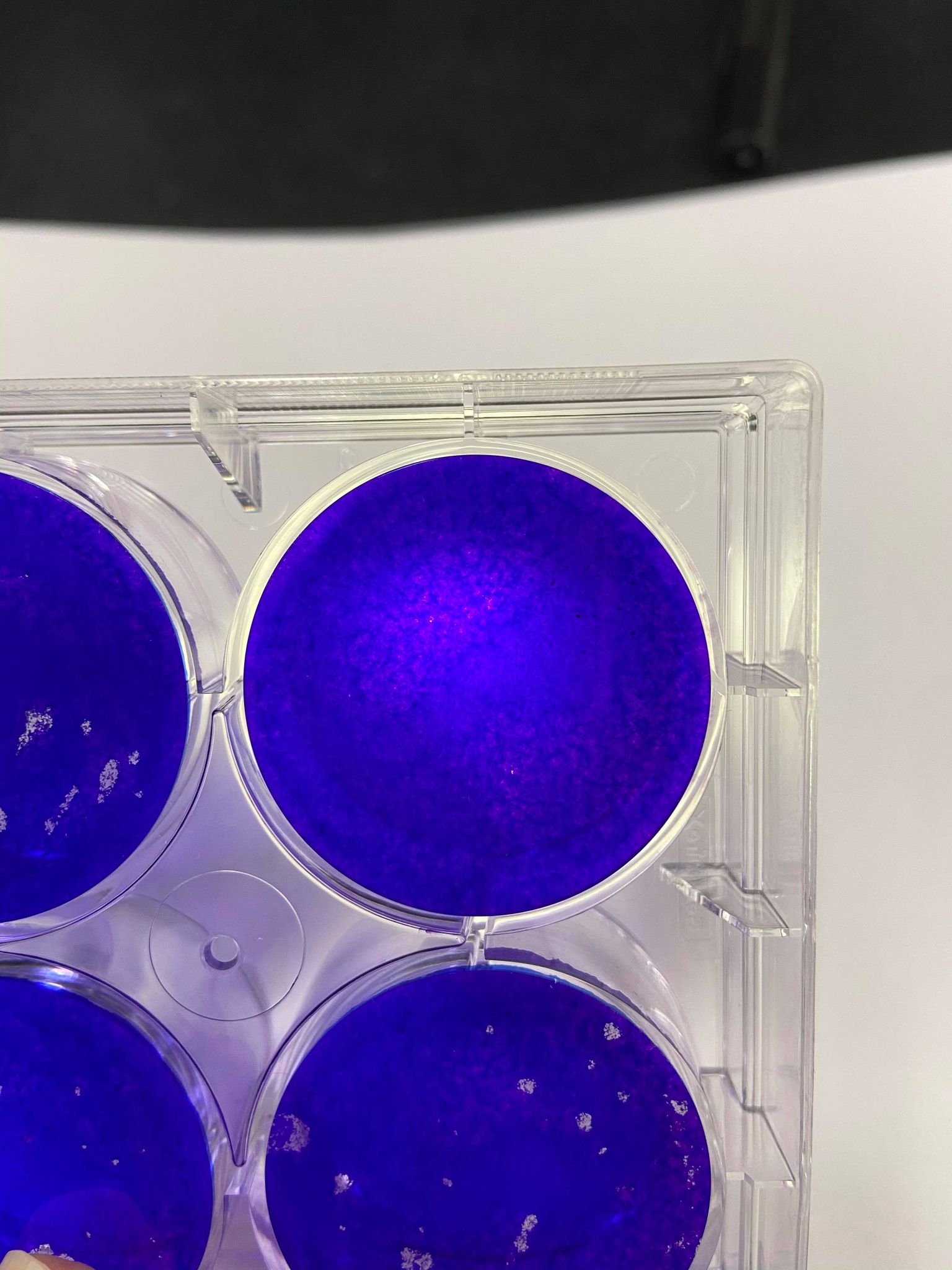

Supplement: Supplementary file 1 [file foods-14-04047-s001.zip › Individual Figures/Figure S2/PLw/Control.jpg]

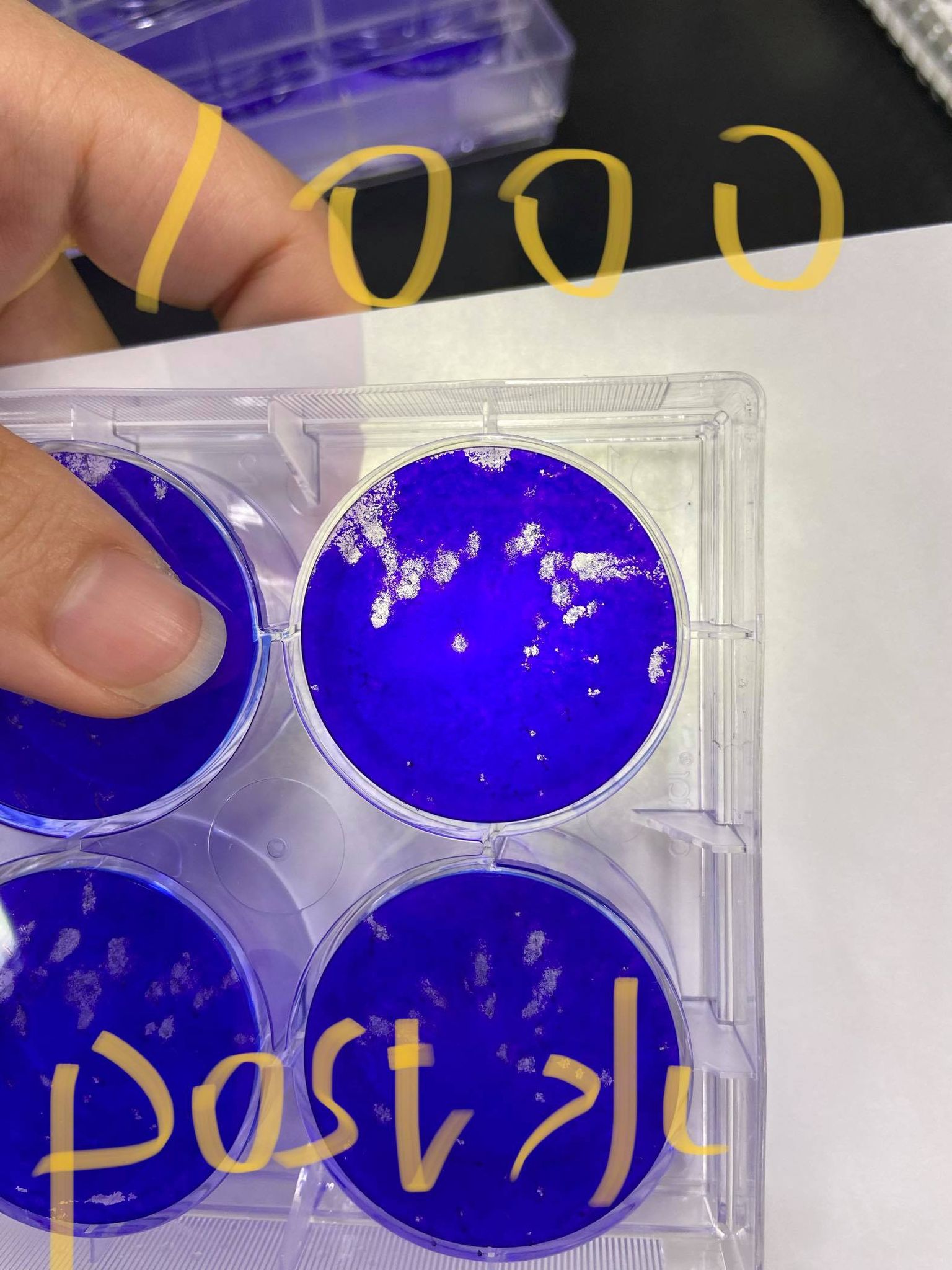

Supplement: Supplementary file 1 [file foods-14-04047-s001.zip › Individual Figures/Figure S2/PLw/Post PLw 1000.jpg]

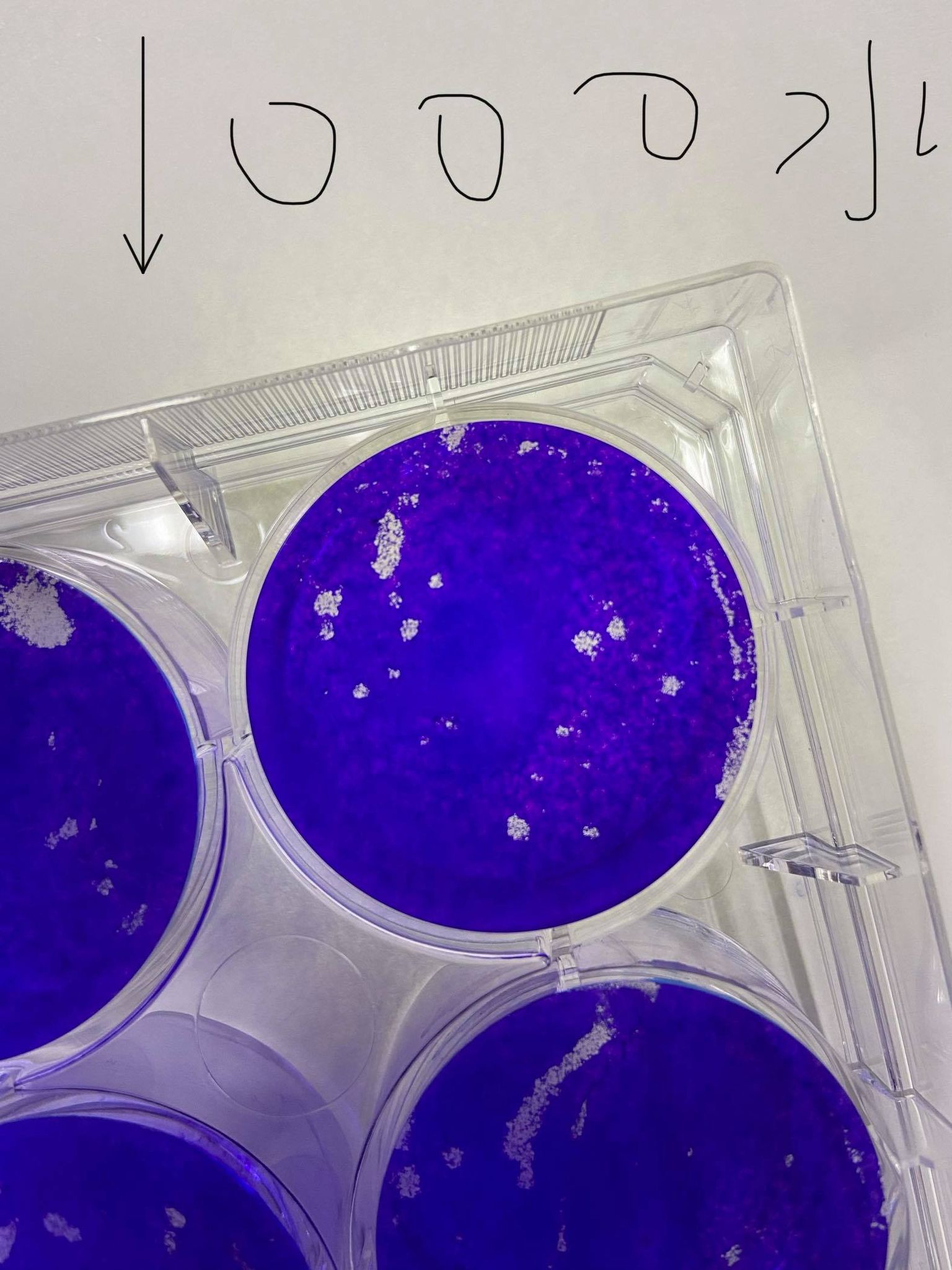

Supplement: Supplementary file 1 [file foods-14-04047-s001.zip › Individual Figures/Figure S2/PLw/Pre PLw 1000.jpg]

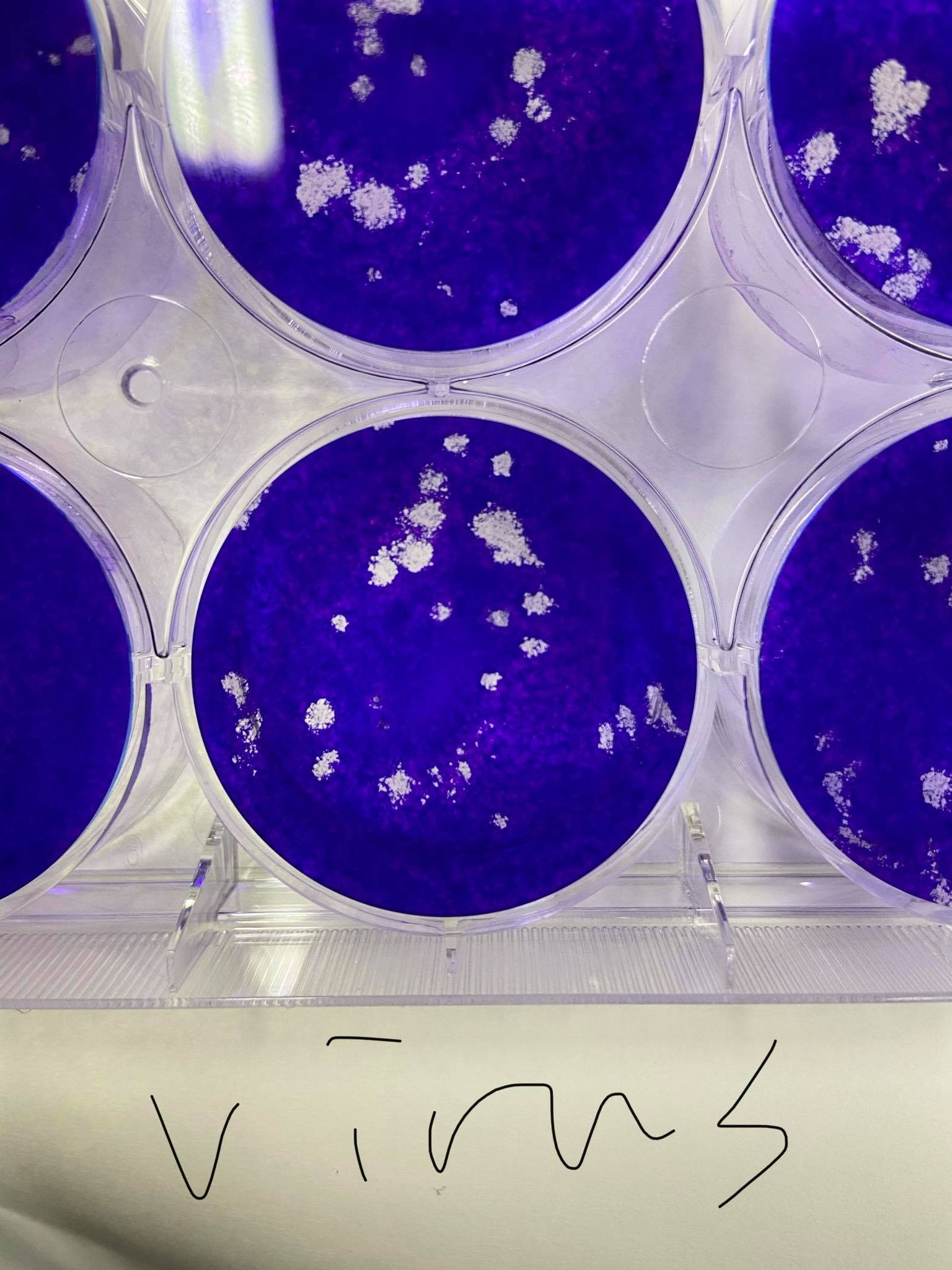

Supplement: Supplementary file 1 [file foods-14-04047-s001.zip › Individual Figures/Figure S2/PLw/Virus PLw.jpg]
